# Agreement Between Acoustic Rhinometry and Computed Tomography Nasal Cross-Sectional Areas Perpendicular to the Direction of the Airflow

**DOI:** 10.3390/diagnostics16020229

**Published:** 2026-01-11

**Authors:** Aris I. Giotakis, Helen Heppt, Matthias Santer, Martin Pillei, Manuel Berger

**Affiliations:** 1 Department of Otorhinolaryngology, Medical University of Innsbruck, 6020 Innsbruck, Austria; arisgiotakis@gmail.com (A.I.G.); helen.heppt@tirol-kliniken.at (H.H.); 2 Department of Industrial Engineering & Management, Management Center Innsbruck, The Entrepreneurial School, 6020 Innsbruck, Austria; martin.pillei@mci.edu; 3 Department of Medical Technologies, Management Center Innsbruck, The Entrepreneurial School, 6020 Innsbruck, Austria; manuel.berger@mci.edu

**Keywords:** acoustic rhinometry, computed tomography, nasal cavity, nasal obstruction, respiratory airflow, case–control studies

## Abstract

**Background/Objectives**: To thoroughly compare acoustic rhinometry (AR) with computed tomography (CT) cross-sectional areas that are approximately perpendicular to the direction of the nasal airflow (CT-CSA). **Methods**: We retrospectively examined subjects scheduled for functional nasal surgery, along with preoperative CT and AR. CT-CSAs were assessed in several nasal planes in the first 5 cm of the nasal airway. Area sizes and distances of the CT-CSAs from the columella served to create a CT curve analogous to the AR curve. AR curves were digitized. We examined the correlation and agreement (using the Bland–Altman method) between CT curves and digitized AR curves, as well as between selected CT-CSAs and the first two-encountered AR minimal cross-sectional areas (AR-MCA_1_ and AR-MCA_2_). Correlation was investigated by univariate analysis of variance and Pearson’s correlation. Agreement was examined by the Bland–Altman method. **Results**: In 33 subjects, the correlation of digitized AR with CT was moderate (r = 0.76; *p* < 0.001). AR, in general, underestimated the actual nasal area by 15%. AR-MCA_1_ and AR-MCA_2_ were closest to the CT-CSA of the nasal valve and the incisive canal, respectively. We noted a mainly moderate correlation between the CT-CSA of the nasal valve and AR-MCA_1_ (all r > 0.59; all *p* < 0.001) in contrast to the weaker correlations between the CT-CSA of the incisive canal and AR-MCA_2_. **Conclusions**: AR may underestimate the actual nasal area by 15%. AR-MCA_1_ and AR-MCA_2_ were closest to the CT-CSA of the nasal valve and the incisive canal, respectively.

## 1. Introduction

Chronic nasal obstruction due to nasal skeletal stenosis is a common symptom encountered by otorhinolaryngologists. Holmstrom and coauthors stated back in 1990 that the development of reliable methods for objective assessment of nasal obstruction has been slow [1]. Indeed, even today, otorhinolaryngologists may express doubt about indication of functional nasal surgical procedures such as septoplasty, despite years of practicing objective assessment methods [2].

Current objective methods have disadvantages that limit their acceptance among otorhinolaryngologists. Active anterior rhinomanometry (RMM) and acoustic rhinometry (AR) are well-known standardized procedures [3,4,5,6]. However, they are not easily available. On the contrary, the availability of computed tomography (CT) in hospitals offers significant advantages. CT can assess the anterior cartilaginous nose without adapter- or nozzle-related tissue distortion [7]. It depicts the exact nasal and paranasal anatomy. It is less error-prone than RMM and AR. It is verifiable and not examiner-dependent. Disadvantages of CT include higher costs compared to AR. Indeed, there might be institutions where CT is not available due to high cost, specialized staffing requirements, and maintenance demands. In such settings, the use of AR may be more feasible. Radiation exposure might be considered an additional disadvantage. Nevertheless, the radiation exposure is considered low [8] or even ultra-low [9]. Widmann and coauthors reported similar target-registration errors of standard baseline and low-dose protocols [8]. Also, Tamminen and coauthors mentioned that even ultra-low-dose radiation may be considered sufficient for surgical planning [9].

Despite the potential of CT in the evaluation of nasal patency, reports about its clinical utility have been conflicting [10,11,12]. Therefore, CT still needs to be compared with standardized methods, which are RMM and AR. Correlation of RMM with CT based on computational fluid dynamics has been found to be moderate, with results being similar yet not the same [13,14]. Furthermore, multiple reports that investigated the correlation of AR with CT cross-sectional areas yielded different results [5,15,16]. This might be due to lack of standardized assessment of CT.

Recently, there have been several attempts to fill this gap with the design of cross-sectional areas (CT-CSAs) that are approximately perpendicular to the direction of the airflow using easily found bony landmarks [17,18,19]. In these studies, the authors described CT-CSAs at eight different planes of the nose, some of which correlated weakly with AR.

In the current study, we intended to examine the correlation and agreement of AR with standardized CT-CSAs approximately perpendicular to the direction of the airflow in more detail. To this end, we used multiple comparison points, based on the variables of area and distance, derived from both techniques.

## 2. Materials and Methods

### 2.1. Study Design and Population

In this retrospective study, subjects who underwent surgery for chronic nasal obstruction at the University Department of Otorhinolaryngology, Head and Neck Surgery, between January 2017 and December 2020 were eligible. Of these, we used the SPSS random sample routine to identify a sex-balanced random sample of 60 subjects. This was drawn for pragmatic reasons, since analysis of CT-CSAs in eight planes for each side of the nose was carried out manually by a single investigator. Of this random sample, subjects were included in the study population if cone beam CT (CBCT) scans and AR were available before surgery. Subjects were excluded if sinus opacification, facial or cephalic dysmorphic syndromes, or facial bone trauma were present.

The study protocol was approved by the ethics committee of the Innsbruck Medical University on 12 December 2019 (1261/2019). The protocol was not limited to the current study; rather, it was part of an umbrella project that should retrospectively examine the use of CT as an objective nasal patency assessment method. Therefore, some of the data of the current study were also collected retrospectively after December 2019.

For thorough comparison between AR and CT, we used multiple comparison points. Usually, AR is compared with CT by some arbitrarily chosen CT-CSA and only two AR parameters, which are the first and second minimal cross-sectional areas (MCA). To obtain more comparison points and compare both techniques thoroughly, we transformed the acoustic rhinometry curve into numerical x-y coordinates via digitization of the curve. Furthermore, to avoid comparison between only the “area” variables, we measured not only the cross-sectional areas in CT but also the distances of each standardized CT planes from the columella. This would serve as an analogous to the “distance” variables of AR, making the comparison between a CT curve and an AR curve more feasible. Moreover, the comparison of the CT curve and the AR curve served to identify which of the CT-CSAs should be compared to the first and second clinically used MCA of AR.

### 2.2. Cone Beam Computed Tomography

The CBCT protocol (KaVo 3D eXam, KaVo, Biberach, Germany) used a slice thickness of 0.3 mm, voxel size 0.3 × 0.3 × 0.3 mm, and 536 × 536 matrix. The software Syngo-share-view (Siemens Healthcare Diagnostics GmbH, Vienna, Austria) was used to visualize the DICOM datasets and to carry out the measurements. Default settings for the window and level (window width: 3200; level: 600) were used. Using multiplane reconstructions, axial, sagittal, and coronal planes were simultaneously visualized in a multi-window display [17,18,19].

### 2.3. CT-CSA Design

The CT-CSA of the right nose and the CT-CSA of the left nose were measured separately in mm^2^. In total, eight CT-CSAs of the nasal airway were defined in the mid-sagittal plane, according to previous studies [17,18,19]. These CT-CSAs were tilted at different angles to the nasal floor. The design of each CT-CSA was based on bony landmarks.

**Figure 1:**
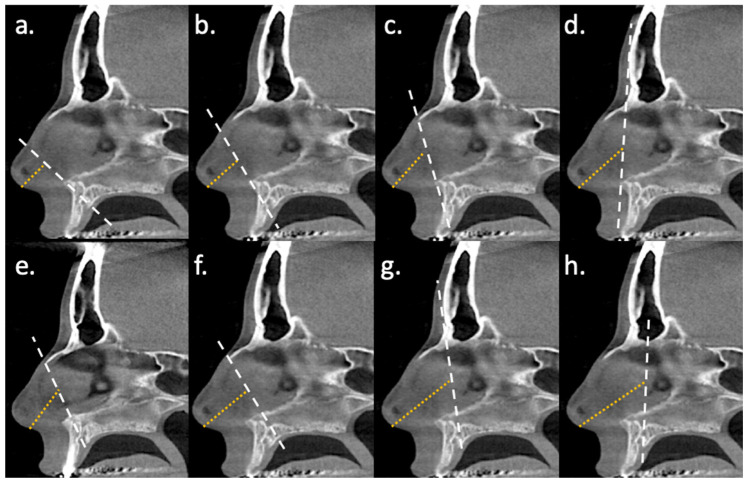
Identification of eight planes perpendicular to the curved airflow in the nose in a mid-sagittal section. The white dashed line indicates the selected oblique plane using as pivot point the anterior nasal spine in (**a**–**d**), the most cranial part of the premaxilla at the level of the anterior borders of the ascending processes of the maxilla in (**e**), and the anterior edge of the superior ostium of the incisive canal in (**f**–**h**). The yellow dashed line indicates the distance from the midline of the caudal border of the columella to the midline of the CT-CA mid-sagittal design. The corresponding cross-sectional areas of the nasal airways are as follows: (**a**) CT-CSA_ant-30_, (**b**) CT-CSA_COT-2_, (**c**) CT-CSA_ant-60_, (**d**) CT-CSA_ant-90_, (**e**) CT-CSA_COT-3_, (**f**) CT-CSA_post-50_, (**g**) CT-CSA_post-80_, and (**h**) CT-CSA_post-100_.

The first CT-CSA was defined by the anterior nasal spine and the posterior edge of the inferior ostium of the incisive canal, which resulted in a CT-CSA tilted about 30° to the nasal floor (CT-CSA_ant-30_). The second CT-CSA was defined by the anterior nasal spine and the anterior edge of the intranasal suture, which resulted in a CT-CSA tilted about 60° to the nasal floor (CT-CSA_ant-60_). The third CT-CSA was defined by the anterior nasal spine and the most ventral part of the frontal bone, which resulted in a CT-CSA tilted about 90° to the nasal floor (CT-CSA_ant-90_) (Figure 1) [17].

The fourth CT-CSA was defined by the anterior nasal spine and a second point. The latter was located at the midpoint of a line along the nasal dorsum, extending from the anterior edge of the intranasal suture (K-area) to its intersection with the line of the CT-CSA_ant-30_. This resulted in a CT-CSA tilted about 45° to the nasal floor. This plane was similar to the plane 2 described by Cottle (CT-CSA_COT-2_) (Figure 1) [19]. The fifth CT-CSA was defined by the anterior edge of the intranasal suture (K-area) and the most cranial edge of the premaxilla at the level of the anterior borders of the ascending processes of the maxilla. This plane corresponded to the area 3 as described by Cottle (CT-CSA_COT-3_) (Figure 1) [19].

The sixth CT-CSA was defined by the anterior edge of the superior ostium of the incisive canal and the anterior edge of the intranasal suture, which resulted in a CT-CSA tilted about 50° to the nasal floor (CT-CSA_post-50_). The seventh CT-CSA was defined by the anterior edge of the superior ostium of the incisive canal and the most ventral part of the frontal bone, which resulted in a CT-CSA tilted about 80° to the nasal floor (CT-CSA_post-80_). The eighth CT-CSA was defined by the anterior edge of the superior ostium of the incisive canal and the posterior edge of the inferior ostium of the incisive canal, which resulted in a CT-CSA tilted about 100° to the nasal floor (CT-CSA_post-100_) (Figure 1) [18].

CT-CSA segmentation was performed manually by a single investigator. The investigator used the drawing polygon function to mark the border between the black space of the nasal airway and the grey area of the surrounding tissue. The area outlined by the border was measured as the CT-CSA [17,18,19].

### 2.4. Distance of CT-CSA from the Columella

To better assess the agreement of CT with AR, we also measured the distance of each of the eight planes from the nasal entrance (Dis-CT), as an analogue value to the distance of the minimal cross-sectional area derived by AR from the nozzle. Dis-CT was assessed in the mid-sagittal plane by measuring the distance (mm) from the midline of the caudal border of the columella to the midline of each mid-sagittal plane (Figure 1). The CT-CSA and Dis-CT would depict CT as a CT curve in the form of a typical AR curve, with the *X*-axis and *Y*-axis corresponding to the cross-sectional areas and their distance from the entrance of the nose, respectively.

### 2.5. Acoustic Rhinometry

For AR, the Rhino-Sys system (Otopront, Hohenstein, Germany) was used. Nasal xylometazoline spray was used routinely as part of the AR examination. Prior to visit, no subject had self-administered nasal xylometazoline spray on the examination day. AR was carried out before and 10 min after decongestion with three puffs (directed to the inferior turbinate, nasal septum, and nasal roof, approximately 180 μL) of nasal xylometazoline spray, 0.05%, per side. All parameters were documented separately for each side of the nose. Only data before decongestion were used for analysis. AR variables included the first (AR-MCA_1_) and second (AR-MCA_2_) minimal cross-sectional areas in mm^2^. AR-MCA_1_ and AR-MCA_2_ data were accompanied by the distances from the nozzle in mm.

### 2.6. Digitization of Acoustic Rhinometry

The cross-sectional areas (CT-CSAs), as well as their distances from the columella (Dis-CT), were analogous to the AR cross-sectional areas and their distance from the nozzle, respectively. However, there were only two AR-MCAs (AR-MCA_1_ and AR-MCA_2_) in contrast to the eight different CT-CSAs. To compare CT and AR more completely, AR curves were digitized in order to obtain more pairs of AR cross-sectional areas and distances.

The AR curves were saved in pdf file format and digitized with WebPlotDigitizer 4.2 software (Automeris; San Francisco, CA, USA). Pixel-based image data were converted to numerical x-y coordinates. The raw data were thresholded with RGB colour for right (255,223,204) ± 10 and left (103,148,198) ± 10 to separate the AR curves. A discretization window of 5 × 5 pixels was chosen. The picture resolution was 847 × 757 pixels. More than 2000 X-Y coordinates per AR curve were saved in csv file format. The X- and Y-axes of the csv file were the same X- and Y-axes as those of the AR. The *X*-axis corresponded to area (mm^2^) and the *Y*-axis to distance (mm).

### 2.7. Outcomes of Comparison Between CT and AR

Initially, we compared the CT curve with the digitized AR curve. Here, we assessed the correlation and agreement between the CT curve and the digitized AR curve via a univariate analysis of variance and the Bland–Altman method, respectively.

Furthermore, to allow for comparison with previous studies, we also examined the correlation of carefully selected CT-CSAs with AR-MCA_1_ or AR-MCA_2_ via Pearson’s correlation and their agreement via the Bland–Altman method.

### 2.8. Data Analysis

Data were analyzed using the SPSS 26.0 statistic package (SPSS Inc., Chicago, IL, USA). Count data were tabulated, and for metric data means, standard deviations and 95% confidence intervals (CI) were calculated. Normality of variables’ distribution was tested with the Shapiro–Wilk test.

Correlations were categorized as strong if r > |0.8|, moderate if |0.8| > r > |0.6|, and weak if r < |0.6|. For agreement, the Bland–Altman method was used [20,21,22]. Here, data were organized per subject, nasal side, and CT-CSA level after being transformed into absolute values. Absolute values of the CT-CSA and AR-MCA were logarithmically transformed. The difference between the logarithm of the AR-MCA and the logarithm of the CT-CSA is equal to the logarithm of the proportional deviation of the AR-MCA from the CT-CSA. Since log10(AR-MCA/CT-CSA) = x is equivalent to AR-MCA/CT-CSA =10x, the AR-MCA/CT-CSA can easily be calculated. We performed an inter-subject examination of agreement per nasal side and CT-CSA level.

#### 2.8.1. Comparison of CT Curve with Digitized AR Curve

Csv data of digitized AR curve were assessed as follows: Each AR curve csv file was opened in excel form. Here, distance values were sorted from the smallest to the largest. Negative distance values were deleted. Distance values were then rounded to 2 decimal points with the “round” function. The file was saved as an excel workbook file and re-opened as an SPSS file. This included two columns, which were an area- and a distance column. As already described, more than 2000 pairs (rows) were available. We intended to reduce these pairs into eight pairs to better compare them with the eight CT planes. Therefore, only the digitized AR-distance values that were equal to the Dis-CT values per each plane were selected for analysis. The latter was performed individually for each subject, resulting in matched and equal digitized AR-distance values and CT-distance values per plane and per subject. The digitized AR-MCA values were aggregated as the mean values based on the eight selected digitized AR-distance values. Finally, the CT-CSA values per CT plane for each subject were added to the file. Duplicate cases were deleted. This resulted in eight digitized AR-MCA values and eight CT-CSA values per curve, i.e., nasal side. These pairs were matched with approximately the same distance from the entrance of the nose.

#### 2.8.2. Comparison of CT-CSA with AR-MCA_1_ or AR-MCA_2_

The CT-CSA of the right noses were compared to the AR-MCA_1_ or AR-MCA_2_ of the right noses, and the CT-CSA of the left noses were compared to the AR-MCA_1_ or AR-MCA_2_ of the left noses. A visual comparison between the CT curve and the AR values, i.e., AR-MCA_1_ and AR-MCA_2_, would indicate which of the eight CT planes should be compared to AR-MCA_1_ or AR-MCA_2_.

## 3. Results

### 3.1. Study Population

During the study period, 1005 patients underwent surgery for chronic nasal obstruction. Of the gender-balanced random sample drawn, 33 subjects fulfilled the inclusion criteria and were included as the study population. Seventeen were men. The median age was 26 years (range: 18–56 years). Septoplasty and functional septorhinoplasty were carried out by 20 and 13 patients, respectively.

### 3.2. Distances of Cross-Sectional Areas from the Entrance of the Nose in CT

As expected, the closest and most distant planes from the columella were the anterior 30°- and the posterior 100° plane, respectively. For the anterior 30° plane, the mean distance ± standard deviation was 14 ± 36 mm (range: 9–24 mm), and for the posterior 100° plane, it was 42 ± 39 mm (range: 36–55 mm) (Table 1). As expected, the Cottle-area-2 plane was closer to the columella (19 ± 28 mm) than the Cottle-area-3 plane (27 ± 28 mm). Similar distances were observed for the anterior 90° and the posterior 50° plane (Table 1). All distances differed significantly from plane to plane (paired samples t-test or Wilcoxon signed ranks test, where appropriate; *p* < 0.001).

### 3.3. Correlation of CT Curve with Digitized AR Curve

**Figure 2:**
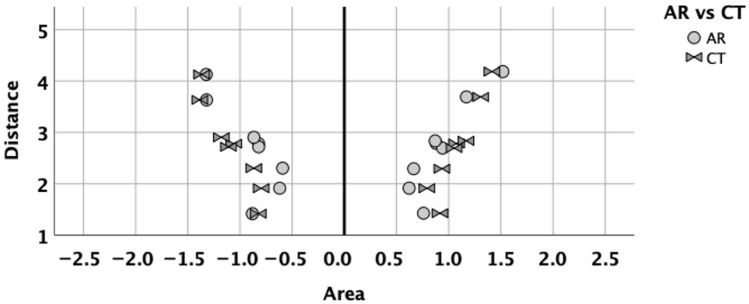
Comparison of digitized AR curve with CT curve. *X*-axis: area in cm^2^. *Y*-axis: distance in cm. Light grey circles and dark grey opposing triangles correspond to AR and CT, respectively. Negative and positive area values correspond to right and left nose, respectively.

A visual comparison of the digitized AR with CT is presented in Figure 2. The correlation of AR with CT was moderate. The correlation coefficient of digitized AR with CT was 0.76 (*p* < 0.001), after removing the variations due to subjects and nasal sides.

The best correlations of CT with digitized AR were observed for the following planes: Cottle-area-2 (all r = 0.58 to 0.74; all *p* ≤ 0.001) and Cottle-area-3 (all r = 0.58 to 0.60; all *p* = 0.001) (Table 2).

### 3.4. Agreement Between CT Curve and Digitized AR Curve

**Figure 3:**
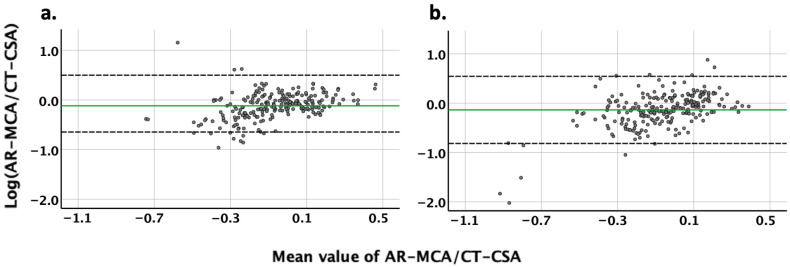
Agreement of the digitized AR curve with CT curve (n = 33) using Bland–Altman plots. AR-MCA: digitized minimal cross-sectional area derived by acoustic rhinometry. CT-CSA: cross-sectional area derived by computed tomography. Y-Axis: difference in logarithmic AR-MCA from logarithmic CT-CSA. X-Axis: mean value of logarithmic AR-MCA and logarithmic CT-CSA. Continuous green horizontal line: mean value of differences. Upper and lower black dashed horizontal line: upper and lower 95% CI of mean value of differences, respectively. On average, the digitized AR-MCA was 24% and 27% smaller than the CT-CSA in the (**a**) right and (**b**) left nose, respectively.

On the right noses, the mean value ± 95% CI of the logarithm of the proportional deviation of the AR-MCA from the CT-CSA was −12% ± 53%. After applying the equations log10(AR-MCA/CT-CSA) = x and AR-MCA/CT-CSA =10x, the AR-MCA on the right noses was found to be 0.76 ± 3.37 times larger (or 1.32 ± 3.37 smaller) than the CT-CSA. Accordingly, on the left noses, the AR-MCA was 0.73 ± 4.80 times larger (or 1.37 ± 4.80 times smaller) than the CT-CSA (Table 3; Figure 3).

### 3.5. Correlation of CT-CSA with AR-MCA_1_ or AR-MCA_2_

**Figure 4:**
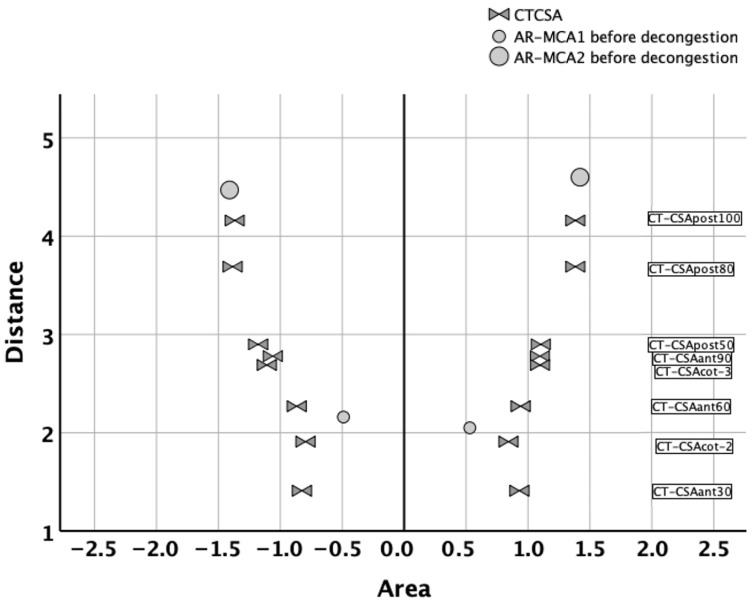
Comparison of cross-sectional areas derived by acoustic rhinometry (AR-MCA_1_ and AR-MCA_2_) with cross-sectional areas derived by CT (CT-CSA). *X*-axis: area in cm^2^. *Y*-axis: distance in cm. Circles and opposing triangles correspond to acoustic rhinometry (AR) and computed tomography (CT), respectively. Negative and positive area values correspond to right and left nose, respectively. Smaller and larger circles correspond to AR-MCA_1_ and AR-MCA_2_, respectively. Each opposing triangle corresponds to the CT-CSA of a specific plane. Starting from below, these are CT-CSA_ant-30_, CT-CSA_COT-2_, CT-CSA_ant-60_, CT-CSA_COT-3_, CT-CSA_ant-90_, CT-CSA_post-50_, CT-CSA_post-80_, and CT-CSA_post-100_. AR-MCA_1_ seemed closest to the CT-CSA of Cottle-area-2 (i.e., nasal valve), while AR-MCA_2_ seemed closest to the CT-CSA_post-100_.

A visual comparison of the CT-CSA with the AR-MCA_1_ and AR-MCA_2_ is presented in Figure 4. Here, AR-MCA_1_ was closest to the Cottle-area-2 (i.e., nasal valve) plane (CT-CSA_COT-2_), and AR-MCA_2_ was closest to the deepest posterior 100° plane (CT-CSA_post-100_). Therefore, AR-MCA_1_ was compared to CT-CSA_COT-2_, and AR-MCA_2_ was compared to CT-CSA_post-100_.

Correlations were somewhat better in the nose anterior to the piriform aperture than in the nose posterior to the piriform aperture. We noted a mainly moderate correlation of CT-CSA_COT-2_ with AR-MCA_1_ (all r > 0.59; all *p* < 0.001) in contrast to the weaker correlations of CT-CSA_post-100_ with AR-MCA_2_ (Table 4).

### 3.6. Agreement Between the CT-CSA and AR-MCA_1_ or AR-MCA_2_

We noted better agreement posterior rather than anterior to the piriform aperture (Table 5). Anterior to the piriform aperture, CT-CSA_COT-2_ was 1.70–1.88 times larger than AR-MCA_1_. Posterior to the piriform aperture, CT-CSA_post-100_ was similar to AR-MCA_2_ (Table 5).

## 4. Discussion

In this retrospective study, we intended to thoroughly examine the association of AR with standardized CT cross-sectional areas in several planes of the nose that are approximately perpendicular to the direction of the airflow. We assumed that the design of these standardized CT-CSAs would allow for a better understanding of the association between AR and CT. AR measures not only cross-sectional areas but also the distance of these cross-sectional areas from the nostril, a variable that few comparison studies have taken advantage of [23,24]. For this reason, we measured the distances of the standardized CT planes from the columella. This helped us create a CT curve that was analogous to the well-known AR curve that is derived from AR Rhino-Sys system (Otopront, Hohenstein, Germany).

This CT curve allowed us to comprehensively compare both techniques using all data after AR curve digitization. Despite the obvious similarity between the AR curve and the CT curve (Figure 2), clinical practice requires the comparison of the CT cross-sectional areas with the traditionally used AR-MCA_1_ or AR-MCA_2_. In the past, CT cross-sectional areas have been arbitrarily chosen for this cause. In the current study, the distances of the CT planes from the columella allowed us to better determine which CT-CSA should be compared to AR-MCA_1_ or AR-MCA_2_ (Figure 4).

The results of this study revealed a moderate correlation between the digitized AR curve and the CT curve (r~0.76; *p* < 0.001), with the digitized AR cross-sectional areas being approximately 15% smaller than the CT cross-sectional areas. This implies that AR, in general, underestimates the actual area of the nasal airway by approximately 15%, considering that CT depicts the actual complex nasal anatomy in contrast to AR, which assumes a simple tube [6]. Interestingly, error rates could be as high as 340%, meaning that AR could be up to 3.4 times smaller or larger than the actual nasal area.

Nevertheless, this might be non-practical information, since only AR-MCA_1_ and AR-MCA_2_ are used in daily practice. For each of these variables, different results were found. The correlation of AR with CT was moderate in the part of the nose anterior to the piriform aperture (all *p* ≤ 0.001) and weak to moderate posterior to the piriform aperture. Moreover, our findings suggested that AR-MCA_1_ and AR-MCA_2_ were closest to the CT-CSA of Cottle-area-2 and CT-CSA_post-100_, respectively (Figure 4).

However, this does not change the fact that AR is more accurate in the anterior nose. The terms “anterior to the piriform aperture” and “posterior to the piriform aperture” still apply for the first 5 cm of the nose (Table 1). This is different than the definition of the “anterior and posterior” nose used in other studies. In these studies, the anterior nose includes MCA_1_ and MCA_2_, which are found in the first 5 cm of the nose, and the posterior nose includes the nose beyond the first 5 cm, where AR is not accurate due to sound loss [25].

Older studies have found similar—but still worse—results when differentiating comparison depending on the anterior and posterior nose [16,23]. Min and Jang found a significant weak correlation of AR with CT in the anterior nose (r = 0.23; *p* < 0.002) but not in the posterior nose (r < 0.15; *p* > 0.2). Here, CT-cross sectional areas were perpendicular to the nasal floor. The authors reported that AR under- and overestimated the actual nasal area in the anterior and posterior nose, respectively [23]. Gilain and coauthors have also found significant correlations of AR with CT cross-sectional areas of the coronal plane in the anterior (r = 0.73; *p* < 0.001) and middle nose (very similar to the posterior nose in the current study; r = 0.56; *p* < 0.005) but not in the posterior nose (*p* > 0.05). Here, the authors did not examine agreement [16]. Better correlations of AR with CT (r = 0.92 to 0.94; *p* < 0.0001) have been observed only in cadaveric studies [5,26]. One possible reason for this is that the size of the airways increases after death [27].

To surpass the side differentiation and compare the CT with AR in a way more relevant to the airflow, we also tried to assign AR to narrow and wide nasal sides. This would result in a comparison of AR with CT, stratified according to narrow sides and wide sides; i.e., AR with CT would be separately compared on the narrow sides and on the wide sides. Therefore, we assigned AR to narrow and wide sides based first on the CT scan and second on the AR itself. However, we noted a discrepancy in the way CT and AR assigned narrow and wide sides (9/33 for CT-CSA_COT-2_ and AR-MCA_1_; 12/33 for CT-CSA_post-100_ and AR-MCA_2_). Considering that CT depicts the exact nasal anatomy, this would imply that AR identified another side as the narrow one 9 (or 12) out of 33 times. Obviously, this could be the result of the nasal cycle since a CT was not performed routinely by every patient after AR. Ideally, each CT should be performed immediately after AR in all subjects systematically. In fact, all subjects received routinely nasal xylometazoline spray, i.e., decongestion, during AR. Therefore, the values of AR after decongestion would originate from a decongested mucosal state in both nasal sides. If every CT would be performed immediately after AR, then the mucosa of both nasal sides in all subjects during CT would remain and be in a decongested mucosal state. This would indicate comparison of AR with CT in a similar mucosal (decongested) state in both nasal sides. The effect of the nasal cycle would be then neutralized, and every CT that was carried out after AR would be unaffected. Both AR and CT examine the anatomy of the nose and are unable to assess functional changes. However, according to our data, 21/33 subjects underwent CT before AR (in a not-decongested mucosal state) and 12/33 after AR (in a decongested state). Only the comparison of AR with CT in subjects that underwent CT after AR (12/33) would be unaffected by the effect of the nasal cycle. In the remaining 21/33 subjects, CT was performed before AR. Usually, the nasal cycle results in the congestion of one nasal side and the decongestion of the other nasal side, and vice versa, in a timely manner. Therefore, in these 21/33 subjects, it was unknown as to which nasal side was congested (or decongested) at the time of AR before decongestion and which nasal side was congested (or decongested) at the time of CT. This would imply that the nasal cycle might have resulted in a different narrow or wide side assignment at the time of AR and CT. Therefore, we abandoned this narrow/wide differentiation due to a high probability of bias.

The current study compared AR with CT in a number of novel ways. We used reproducible CT-CSAs with easily found bony landmarks that are perpendicular to the direction of the nasal airflow. We introduced the distances of each CT plane from the nostril. We digitized the AR curve and compared carefully selected CT-CSAs with AR-MCA_1_ and AR-MCA_2_. The digitization of rhinometric procedures with the WebPlotDigitizer 4.2 software (Automeris; San Francisco, California, USA) has successfully been achieved by Berger and coauthors [14]. Furthermore, a large number of subjects suffering from chronic nasal obstruction due to nasal skeletal stenosis have been investigated.

Some limitations of this study should be mentioned. The distances of the CT planes from the columella were measured, resembling AR distances. However, a degree of discrepancy in the way that the distances were determined in CT and AR (angle, starting point, ending point) cannot be excluded. Therefore, the CT curve and digitized AR curve pairs might not be entirely matched. Furthermore, the intention, for pragmatic reasons, to examine 60 subjects from a pool of 1005 subjects, as well as the final examination of 33 subjects due to eligibility criteria, might have introduced selection bias, even if a random sampling routine was applied. This would limit the generalizability of the findings.

Moreover, a CT was not performed routinely by every patient after AR. As mentioned above, some patients (21/33) underwent CT in a non-decongested mucosal state and others (12/33) in a decongested state. This suggested that the factor nasal cycle was not entirely neutralized since it could have been active in 21/33 subjects. This would affect the comparison of the areas in AR (i.e., AR-MCA) and CT (i.e., CT-CSA). Ideally, each CT should have been performed immediately after AR in all subjects systematically. This would allow for an AR-CT comparison that would be unaffected by the nasal cycle.

Lastly, there seemed to be a discrepancy between the weak-to-moderate correlation and the seemingly perfect agreement of AR-MCA_2_ with CT-CSA_post-100_. Usually, two sets of observations that are highly correlated may have poor agreement. On the contrary, if the two sets of values agree, a high correlation is expected [28]. In the current study, one should not neglect that the seemingly perfect agreement (0.97–1.11) was a mean value with large error rates (up to 60%). Also, we observed similar error rates for the agreement between CT and AR anterior to the piriform aperture. Nevertheless, it should be noted that the purpose of this study was academic; therefore, the findings anterior and posterior to the piriform aperture should be treated as such. The current study did not intend to develop a method to be utilized for individual diagnostic use.

## 5. Conclusions

This study provided new perspectives in our understanding of AR. Our data indicated that AR, in general, underestimated the actual nasal area by 15%. AR-MCA_1_ seemed closest to the CT-CSA of Cottle-area-2 (i.e., nasal valve), while AR-MCA_2_ seemed closest to CT-CSA_post-100_.

## Figures and Tables

**Table 1:** Distances in mm of cross-sectional areas from the entrance of the nose in CT (n = 33).

Plane	Mean	Standard Deviation	Median	Minimum	Maximum
Ant-30	14.3	36.3	13.7	9.0	24.0
Cottle-area-2	19.2	28.1	18.8	14.8	27.3
Ant-60	22.8	26.7	22.2	18.4	30.9
Ant-90	27.9	27.6	28.0	23.6	34.9
Cottle-area-3	26.9	28.0	26.6	21.9	34.1
Post-50	29.1	26.6	28.6	24.5	37.0
Post-80	37.0	28.1	37.4	33.5	45.3
Post-100	41.8	38.7	42.1	35.7	54.5

**Table 2:** Correlation of the CT curve with the digitized acoustic rhinometry curve depending on the CT plane (equal to the AR plane; n = 33).

Nose	Right		Left	
	r ^1^	*p* ^2^	r ^1^	*p* ^2^
Ant-30	0.32	0.081	0.31	0.082
Cottle-area-2	0.74 ^3^	<0.001	0.58	0.001
Ant-60	−0.09	>0.2	−0.07	>0.2
Ant-90	0.09	>0.2	−0.25	>0.2
Cottle-area-3	0.60	0.001	0.58	0.001
Post-50	0.60	<0.001	0.45	0.017
Post-80	0.48	0.013	0.10	>0.2
Post-100	0.57	0.002	−0.14	>0.2

^1^ Correlation, ^2^ *p*-value. ^3^ On the right noses of all patients, correlation of the CT curve with the digitized AR curve at the Cottle-area-2 plane (i.e., nasal valve) and the corresponding AR plane was r = 0.74 (*p* < 0.001).

**Table 3:** Agreement between the CT curve and the digitized acoustic rhinometry curve.

	N	Logarithmic Proportional Deviation % ^1^	AR-MCA/ CT-CSA ^1^
Right	33	−12 ± 53	0.76 ± 3.37
Left	33	−14 ± 68	0.73 ± 4.8

^1^ Mean ± 95% CI from the Bland–Altman agreement measurements; e.g., on the right noses of all subjects, the mean logarithm of the proportional deviation of the digitized AR-MCA from the CT-CSA was −12% ± 53% for 33 measurements. This implied that the digitized AR-MCA was 0.76 ± 3.37 times larger (or 1.32 ± 3.37 times smaller) than the CT-CSA.

**Table 4:** Correlation of CT-CSA with AR-MCA_1_ or AR-MCA_2_ (n = 33).

	CT-CSA_COT-2_/AR-MCA_1_		CT-CSA_post-100_/AR-MCA_2_	
	r ^1^	*p* ^2^	r ^1^	*p* ^2^
Right	0.70 ^3^	<0.001	0.54	0.001
Left	0.59	<0.001	−0.21	>0.2

^1^ Pearson’s correlation. ^2^ *p*-value. ^3^ On the right noses, the correlation of the CT-CSA of Cottle-area-2 (CT-CSA_COT-2_) with the AR-MCA_1_ was r = 0.70 (*p* < 0.001).

**Table 5:** Agreement between CT-CSA and AR-MCA_1_ or AR-MCA_2_ (n = 33).

	CT-CSA_COT-2_/AR-MCA_1_		CT-CSA_post-100_/AR-MCA_2_	
	% ^1^	Abs ^2^	% ^1^	Abs ^2^
Right	28 ± 37 ^3^	1.88 ± 2.36	4.6 ± 45	1.11 ± 2.82
Left	23 ± 47	1.70 ± 2.95	−1.3 ± 58	0.97 ± 3.87

^1^ Logarithmic proportional deviation. ^2^ Absolute value. ^3^ Mean ± 95% CI from the Bland–Altman agreement measurements; e.g., on the right noses of all subjects, the mean logarithm of the proportional deviation of the CT-CSA of Cottle-area-2 (i.e., nasal valve) from AR-MCA_1_ was 28% ± 37% for 33 measurements. This implied that the CT-CSA of Cottle-area-2 (i.e., nasal valve) was 1.88 ± 2.36 times larger than AR-MCA_1_.

## Data Availability

Data used in this study can be requested from the corresponding author upon reasonable request. The data are not publicly available due to privacy restrictions.

## Abbreviations

The following abbreviations are used in this manuscript:

CT	Computed tomography
CT-CSA	Computed tomography cross-sectional areas
AR	Acoustic rhinometry
AR-MCA_1_	First encountered minimal cross-sectional area in acoustic rhinometry
AR-MCA_2_	Second encountered minimal cross-sectional area in acoustic rhinometry
CBCT	Cone beam computed tomography
CI	Confidence intervals
SD	Standard deviation
Dis-CT	Distance of CT-CSA from the columella
CT-CSA_ant-30_	Anterior CT-CSA tilted about 30° to the nasal floor
CT-CSA_COT-2_	CT-CSA of Cottle-area-2 (nasal valve)
CT-CSA_ant-60_	Anterior CT-CSA tilted about 60° to the nasal floor
CT-CSA_ant-90_	Anterior CT-CSA tilted about 90° to the nasal floor
CT-CSA_COT-3_	CT-CSA of Cottle-area-3
CT-CSA_post-50_	Posterior CT-CSA tilted about 50° to the nasal floor
CT-CSA_post-80_	Posterior CT-CSA tilted about 80° to the nasal floor
CT-CSA_post-100_	Posterior CT-CSA tilted about 100° to the nasal floor

## References

[1] Holmstrom M., Scadding G.K., Lund V.J., Darby Y.C. (1990). Assessment of nasal obstruction. A comparison between rhinomanometry and nasal inspiratory peak flow. Rhinology.

[2] Ottaviano G., Fokkens W.J. (2016). Measurements of nasal airflow and patency: A critical review with emphasis on the use of peak nasal inspiratory flow in daily practice. Allergy.

[3] Clement P.A. (1984). Committee report on standardization of rhinomanometry. Rhinology.

[4] Scadding G., Hellings P., Alobid I., Bachert C., Fokkens W., van Wijk R.G., Gevaert P., Guilemany J., Kalogjera L., Lund V. (2011). Diagnostic tools in Rhinology EAACI position paper. Clin. Transl. Allergy.

[5] Hilberg O., Jackson A.C., Swift D.L., Pedersen O.F. (1989). Acoustic rhinometry: Evaluation of nasal cavity geometry by acoustic reflection. J. Appl. Physiol. (1985).

[6] Jackson A.C., Butler J.P., Millet E.J., Hoppin F.G., Dawson S.V. (1977). Airway geometry by analysis of acoustic pulse response measurements. J. Appl. Physiol. Respir..

[7] Riechelmann H., Karow E., DiDio D., Kral F. (2010). External nasal valve collapse—A case-control and interventional study employing a novel internal nasal dilator (Nasanita). Rhinology.

[8] Widmann G., Fasser M., Schullian P., Zangerl A., Puelacher W., Kral F., Riechelmann H., Jaschke W., Bale R. (2012). Substantial dose reduction in modern multi-slice spiral computed tomography (MSCT)-guided craniofacial and skull base surgery. Rofo.

[9] Tamminen P., Jarnstedt J., Numminen J., Lehtinen A., Lehtimaki L., Rautiainen M., Kivekas I. (2023). Ultra-low-dose CBCT: New cornerstone of paranasal sinus imaging. Rhinology.

[10] Cho G.S., Kim J.H., Jang Y.J. (2012). Correlation of nasal obstruction with nasal cross-sectional area measured by computed tomography in patients with nasal septal deviation. Ann. Otol. Rhinol. Laryngol..

[11] Ardeshirpour F., McCarn K.E., McKinney A.M., Odland R.M., Yueh B., Hilger P.A. (2016). Computed tomography scan does not correlate with patient experience of nasal obstruction. Laryngoscope.

[12] Sedaghat A.R., Kieff D.A., Bergmark R.W., Cunnane M.E., Busaba N.Y. (2015). Radiographic evaluation of nasal septal deviation from computed tomography correlates poorly with physical exam findings. Int. Forum Allergy Rhinol..

[13] Cherobin G.B., Voegels R.L., Pinna F.R., Gebrim E., Bailey R.S., Garcia G.J.M. (2021). Rhinomanometry Versus Computational Fluid Dynamics: Correlated, but Different Techniques. Am. J. Rhinol. Allergy.

[14] Berger M., Giotakis A.I., Pillei M., Mehrle A., Kraxner M., Kral F., Recheis W., Riechelmann H., Freysinger W. (2021). Agreement between rhinomanometry and computed tomography-based computational fluid dynamics. Int. J. Comput. Assist. Radiol. Surg..

[15] Mamikoglu B., Houser S., Akbar I., Ng B., Corey J.P. (2000). Acoustic rhinometry and computed tomography scans for the diagnosis of nasal septal deviation, with clinical correlation. Otolaryngol. Head. Neck Surg..

[16] Gilain L., Coste A., Ricolfi F., Dahan E., Marliac D., Peynegre R., Harf A., Louis B. (1997). Nasal cavity geometry measured by acoustic rhinometry and computed tomography. Arch. Otolaryngol. Head. Neck Surg..

[17] Giotakis A.I., Widmann G., Mallien E., Riechelmann F., Heppt H., Riechelmann H. (2023). CT analysis of the anterior nasal airway based on the direction of nasal airflow in patients with nasal obstruction and trauma controls. Eur. Arch. Otorhinolaryngol..

[18] Heppt H., Widmann G., Riechelmann F., Runge A., Riechelmann H., Giotakis A.I. (2024). CT comparison of the nasal airway anterior and posterior to the piriform aperture in patients with and without nasal obstruction. Head. Face Med..

[19] Heppt H., Widmann G., Santer M., Riechelmann F., Riechelmann H., Giotakis A.I. (2025). Comparison of Cottle-Area-2 and Cottle-Area-3 in Computed Tomography Scans of Patients with Nasal Obstruction and Controls. Diagnostics.

[20] Bland J.M., Altman D.G. (1986). Statistical methods for assessing agreement between two methods of clinical measurement. Lancet.

[21] Bland J.M., Altman D.G. (1999). Measuring agreement in method comparison studies. Stat. Methods Med. Res..

[22] Giavarina D. (2015). Understanding Bland Altman analysis. Biochem. Med..

[23] Min Y.G., Jang Y.J. (1995). Measurements of cross-sectional area of the nasal cavity by acoustic rhinometry and CT scanning. Laryngoscope.

[24] Terheyden H., Maune S., Mertens J., Hilberg O. (2000). Acoustic rhinometry: Validation by three-dimensionally reconstructed computer tomographic scans. J. Appl. Physiol. (1985).

[25] Hilberg O. (2002). Objective measurement of nasal airway dimensions using acoustic rhinometry: Methodological and clinical aspects. Allergy.

[26] D’Urzo A.D., Lawson V.G., Vassal K.P., Rebuck A.S., Slutsky A.S., Hoffstein V. (1987). Airway area by acoustic response measurements and computerized tomography. Am. Rev. Respir. Dis..

[27] Guilmette R., Wicks J., Wolff R., Mauderly J. (1992). In Vivo Measurement of Human Nasal Airway Dimensions.

[28] Ranganathan P., Pramesh C.S., Aggarwal R. (2017). Common pitfalls in statistical analysis: Measures of agreement. Perspect. Clin. Res..

